# Mutations of genes in synthesis of the carotenoid precursors of ABA lead to pre-harvest sprouting and photo-oxidation in rice

**DOI:** 10.1111/j.1365-313X.2008.03411.x

**Published:** 2008-04-01

**Authors:** Jun Fang, Chenglin Chai, Qian Qian, Chunlai Li, Jiuyou Tang, Lei Sun, Zejun Huang, Xiaoli Guo, Changhui Sun, Min Liu, Yan Zhang, Qingtao Lu, Yiqin Wang, Congming Lu, Bin Han, Fan Chen, Zhukuan Cheng, Chengcai Chu

**Affiliations:** 1 State Key Laboratory of Plant Genomics and National Centre for Plant Gene Research (Beijing), Institute of Genetics and Developmental Biology, Chinese Academy of Sciences (CAS) Beijing 100101, China; 2 Graduate University of the CAS Beijing 100039, China; 3 State Key Laboratory of Rice Biology, China National Rice Research Institute, Chinese Academy of Agricultural Sciences Hangzhou 310006, China; 4 Centre for Biological Electron Microscopy, Institute of Biophysics CAS, Beijing 100101, China; 5 State Key Laboratory of Photosynthesis and Environmental Molecular Physiology, Institute of Botany CAS, Beijing 100093, China; 6 National Centre for Gene Research CAS, Shanghai 200233, China; 7 Laboratory of Molecular and Developmental Biology, Institute of Genetics and Developmental Biology CAS, Beijing 100101, China

**Keywords:** rice, pre-harvest sprouting, photo-oxidation, carotenoid biosynthesis, abscisic acid

## Abstract

Pre-harvest sprouting (PHS) or vivipary in cereals is an important agronomic trait that results in significant economic loss. A considerable number of mutations that cause PHS have been identified in several species. However, relatively few viviparous mutants in rice (*Oryza sativa* L.) have been reported. To explore the mechanism of PHS in rice, we carried out an extensive genetic screening and identified 12 PHS mutants (*phs*). Based on their phenotypes, these *phs* mutants were classified into three groups. Here we characterize in detail one of these groups, which contains mutations in genes encoding major enzymes of the carotenoid biosynthesis pathway, including phytoene desaturase (OsPDS), ζ-carotene desaturase (OsZDS), carotenoid isomerase (OsCRTISO) and lycopene *β*-cyclase (β-OsLCY), which are essential for the biosynthesis of carotenoid precursors of ABA. As expected, the amount of ABA was reduced in all four *phs* mutants compared with that in the wild type. Chlorophyll fluorescence analysis revealed the occurrence of photoinhibition in the photosystem and decreased capacity for eliminating excess energy by thermal dissipation. The greatly increased activities of reactive oxygen species (ROS) scavenging enzymes, and reduced photosystem (PS) II core proteins CP43, CP47 and D1 in leaves of the *Oscrtiso*/*phs3-1* mutant and *OsLCY* RNAi transgenic rice indicated that photo-oxidative damage occurred in PS II, consistent with the accumulation of ROS in these plants. These results suggest that the impairment of carotenoid biosynthesis causes photo-oxidation and ABA-deficiency phenotypes, of which the latter is a major factor controlling the PHS trait in rice.

## Introduction

The phenomenon of germination of cereal grains in the ear or panicle, usually under wet conditions shortly before harvest, is termed pre-harvest sprouting (PHS) or vivipary. Pre-harvest sprouting of cereal grains not only causes reduction of grain yield but also affects the quality of the grain. In Southeast Asia, PHS frequently occurs in rice due to the long spell of rainy weather in early summer and autumn (Wan *et al.*, 2006). In south China alone, heavy PHS sometimes occurs in >6% of the rice acreage, which could be up to 20% for hybrid rice (Guo *et al.*, 2004).

Previous work demonstrated that mutants impaired in abscisic acid (ABA) biosynthesis or responsiveness, such as maize viviparous (*vp*), Arabidopsis ABA-deficient (*aba*) and ABA-insensitive (*abi*) mutants, often produce precociously germinating seeds (McCarty, 1995). At least 10 viviparous mutants have been identified from maize (*Zea mays* L.), most of which (*vp2*, *vp5*, *vp7*, *vp9*, *w3*, *y3*, and *y9*) were blocked in biosynthesis of the carotenoid precursors for *de novo* ABA synthesis (Figure S1; Singh *et al.*, 2003). In the early steps of carotenoid biosynthesis, the head-to-head condensation of C_20_ geranylgeranyl diphosphate (GGPP) molecules to produce a C_40_ carotenoid phytoene (colorless) is mediated by a soluble enzyme called phytoene synthase (PSY), which is the first committed step in carotenoid synthesis. Subsequently the phytoene undergoes four desaturation reactions with the production of lycopene (Cunningham and Gantt, 1998). Then, a series of steps including cyclization and hydroxylation reactions take place to yield α-carotene, β-carotene, lutein, xanthophyll, and zeaxanthin. The C_40_ carotenoid precursor is cleaved and followed by a two-step conversion of the intermediate xanthoxin to ABA via ABA aldehyde (Schwartz *et al.*, 2003; Xiong and Zhu, 2003).

The maize *Vp5* gene was found to encode a phytoene desaturase (PDS), and transgenic rice plants harboring the *PDS*–RNAi construct showed a clear albino phenotype (Hable *et al.*, 1998; Miki and Shimamoto, 2004). The maize *vp9* mutant and the *non dormant-1* (*nd-1*) mutant of sunflower (*Helianthus annuus* L.) have mutations in the gene coding for zeta-carotene desaturase (ZDS; Conti *et al.*, 2004; Matthews *et al.*, 2003). The carotenoid isomerase (CRTISO) gene was cloned from the *tangerine* mutant of tomato and *ccr2* mutant of Arabidopsis (Isaacson *et al.*, 2002; Park *et al.*, 2002). The *Vp7*/*Ps1* gene encodes a lycopene-β- cyclase and is necessary for the accumulation of both ABA and carotenoid zeaxanthin in mature maize embryos; the mutant is easily discernible as it has pink kernels because of lycopene accumulation (Singh *et al.*, 2003). These mutants containing defects in carotenoid precursor synthesis exhibit pleiotropic phenotypes, such as albino or pale green, non-viable seedlings and vivipary, due to deficiencies of carotenoid and ABA.

Within the thylakoid membranes of chloroplasts, carotenoids are found to be bound to specific protein complexes of photosystem I (PS I) and photosystem II (PS II), where they augment the light-harvesting capacity by absorbing light in the blue–green range of the visible spectrum (450–550 nm) and transferring the energy to chlorophylls (Holt *et al.*, 2005; Moise *et al.*, 2005). Carotenoid deficiency often causes aberrations in plastid ultrastructure, such as etioplasts from the cotyledon of dark-grown *ccr2* mutants (carotenoid isomerase, *CRTISO* mutation) which lack prolamellar bodies (PLBs; Park *et al.*, 2002). In addition, carotenoids play an essential role in photoprotection in plants. During photosynthesis, the excess absorbed energy can be eliminated as heat by de-exciting ^1^Chl through the process of non-photochemical quenching of chlorophyll fluorescence (NPQ), minimizing the generation of harmful reactive oxygen species (ROS; Ma *et al.*, 2003; Niyogi, 1999; Tracewell *et al.*, 2001).

Besides, as accessory pigments in photosynthesis and photoprotectors preventing photo-oxidative damage, carotenoids can also be the precursors to the hormone ABA. Analysis of mutant and transgenic plants has provided strong evidence that ABA biosynthesis and responses to this phytohormone are clearly involved in the onset and maintenance of dormancy and inhibition of PHS (Bewley, 1997). Abscisic acid biosynthetic mutants fail to induce seed dormancy and exhibit a vegetative wilty phenotype. These phenotypes have been used to define and prove the function of ABA in seed dormancy and water relations. The seeds of ABA-deficient mutants of tomato (*Solanum lycopersicum* L., e.g. *sitiens*), Arabidopsis (e.g. *aba*) and maize treated with fluridone (a carotenoid biosynthesis inhibitor) germinate readily when placed in water and sometimes show vivipary (Fong *et al.*, 1983; Groot and Karssen, 1992; Leon-Kloosterziel *et al.*, 1996a).

Another phytohormone, gibberellin (GA), is also involved in the release from dormancy of various species. It has been shown that GA-deficient mutants of Arabidopsis and tomato are dependent on exogenous GA for germination (Koornneef *et al.*, 2002). Further work in maize showed that inhibition of GA biosynthesis mimics the effect of exogenous ABA in suppressing vivipary (White and Rivin, 2000; White *et al.*, 2000). These results suggested that the ABA/GA ratio and not the absolute hormone content controls germination (Finch-Savage and Leubner-Metzger, 2006).

In contrast to the intensive molecular and genetic studies of seed dormancy in maize and Arabidopsis, the molecular mechanism of seed dormancy in rice is poorly understood, mainly because of the lack of available mutants with reduced dormancy. So far most research has focused on the quantitative trait locus (QTL) analysis of the natural differences in seed dormancy characteristics (Gonzalez-Guzman *et al.*, 2004; Gu *et al.*, 2004, 2006; Takeuchi *et al.*, 2003; Wan *et al.*, 2006). Only a few reports have been published on *phs* mutants and only one gene (*OsABA1*) has been cloned which was involved in ABA biosynthesis (Agrawal *et al.*, 2001).We performed a large-scale screening of a rice T-DNA/Tos17 insertion mutant population to identify the genes involved in PHS. The warm and damp weather, a good elicitor of PHS, during the harvest season in Zhejiang Province on the southeast coast of China, enables us to isolate *phs* mutants efficiently. In an intensive screening of approximately 16 000 rice T_1_ mutant lines, we obtained 12 viviparous mutants. In this paper, four genes involved in carotenoid precursors of ABA biosynthesis were cloned. Our results suggested that the impairment in synthesis of the carotenoid precursors of ABA leads to photo-oxidation and PHS in rice, which will definitely be helpful for elucidating the molecular mechanisms of PHS in other crops such as wheat and barley that are susceptible to PHS.

## Results

### Identification and genetic analysis of the rice phs mutants

To identify rice *phs* mutants, we have screened a T-DNA/Tos17-mutagenized population (Nipponbare background) under field conditions in Hangzhou, downstream of the Yangtze River with a relatively high degree of humidity. Approximately 16 000 transgenic T_1_ lines were screened prior to harvest by visual inspection in the paddy field, and 27 putative mutants were identified with a viviparous phenotype. A representative mutant is shown in Figure S2(a). T_2_ seeds of these 27 putative *phs* mutants were then grown in Beijing with a lower degree of humidity for a second round of screening. From the secondary screening, 12 mutants showing a viviparous phenotype were recovered (Figure S2b), which could be simply categorized into three groups based on phenotypes besides vivipary. Mutants from category I exhibit an albino or photobleaching phenotype (Figure S2c); while mutants from categories II and III do not show an albino or photobleaching phenotype but have an enhanced wilty phenotype under conditions of water stress (category II; Figure S2d) or with embryo/seedling-lethal phenotypes (category III; Figure S2e). We present here a detailed characterization of six mutants which belong to category I (Table 1).

**Table 1:** Rice pre-harvest sprouting mutants and corresponding genes

Category	Original line	Gene	Arabidopsis homolog	Mutation	Chromosome location	Protein (a.a.)	Accession no.
I	T01	*OsPDS*	AT4G14210	Insertion	Chr.3, 20.3cM	566	LOC_Os030g08570
	HF807	*OsZDS*	AT3G04870	Insertion	Chr.7, 41.7cM	578	AK065213
	HG4123			Deletion			
	T09	OsCRTISO*	AT1G06820	Substitution	Chr.11, 84.6cM	586	EF417892
	HC2621	β-*OsLYC*	AT3G10230	Deletion	Chr.2, 23.3cM	493	OC_Os02g09750
	HD1449			Deletion			

* The nucleotide sequence of *OsCRTISO* reported in this paper has been submitted to GenBank under accession number EF417892.

**Figure 1:**
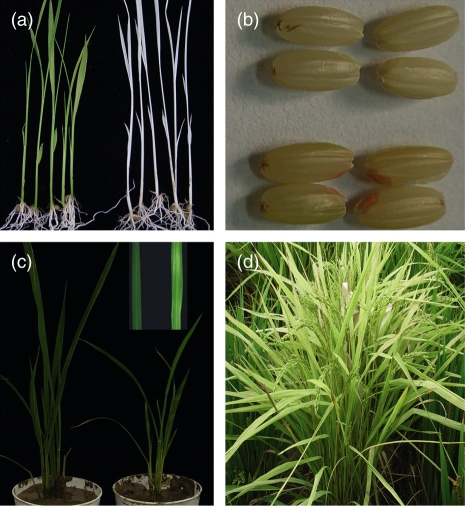
The phenotypes of pre-harvest sprouting (*phs*) mutants in category I.
The albino phenotype of *phs* seedlings.The pink-embryo seeds of *phs4-1* and *phs4-2* lines.Phenotypes of the wild type (left) and *phs3-1* mutant (right) at the early tillering stage. The inset shows the magnified leaves of the wild type and *phs3-1* mutant, respectively.The *phs3-1* plant shows pale green over the whole leaves at the mature stage in the field.

To further characterize these mutants the viviparous seedlings were rescued, and five viviparous mutant lines showed albino seedlings (Figure 1a), these homozygous plants eventually died at about 4 weeks after germination. Interestingly, the homozygous seeds of two mutant lines among them had pink embryos (Figure 1b). Moreover, one of the viviparous mutant lines, T09, developed alternating green and yellow crossbands on the leaf blades at the tillering stage (Figure 1c), like rice *zebra* mutants previously described (Kusumi *et al.*, 2000), and finally showed pale green on entire plants when mature (Figure 1d).

Except for the T09 mutant, all the other viviparous mutants (T_1_ plants) are lethal due to lack of pigments, therefore only two genotypes – heterozygous and wild type in the T_1_ mutant seeds – could germinate after sowing. Statistical analysis of segregation ratios of viviparous and non-viviparous plants demonstrated that the segregation of viviparous and non-viviparous plants is in line with the expected 2:1 ratio (Table S2). Furthermore, in T_2_ progeny (seeds in the ear) obtained from the viviparous plants, the mutant phenotype segregated in 3:1 (Wt: vivipary, data not shown), indicating that each mutant phenotype was caused by a single recessive mutation. Subsequently genetic analysis suggested that these six mutants could be assigned to four loci and were consecutively designated as *phs1* through *phs4*, respectively, of which two mutant alleles were identified in *phs2* (*phs2-1* and *phs2-2*) and *phs4* (*phs4-1* and *phs4-2*; see Table 1). In addition, we later identified another two *phs3* alleles by the *zebra* phenotype (see below).

### Molecular cloning of PHS1 through PHS4 genes

Genetic analysis suggested that T-DNA was not co-segregated with the mutant phenotype in all these mutants (data not shown). We then made a rough mapping to identify candidate genes in these mutants, and the *PHS* genes were located to different chromosomes (Table 1 and Figure S5).

**Figure 2:**
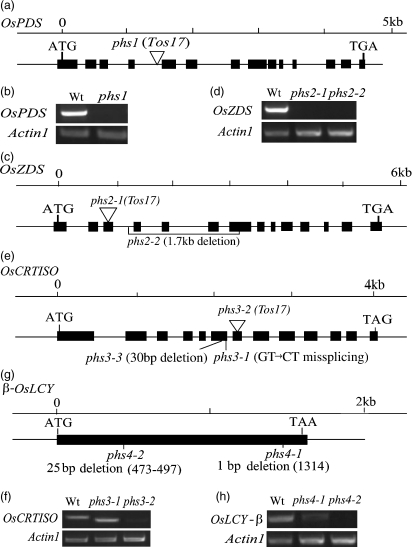
The sites of mutation and the expression of the pre-harvest sprouting (*PHS*) genes.
The position of the mutation in the *OsPDS* gene of *phs1* mutant.Expression of the *OsPDS* gene in the wild type and mutant.The positions of the mutation in the *OsZDS* gene of *phs2-1* and *phs2-2* mutants.Expression of the *OsZDS* gene in the wild type and mutants.The positions of the mutation in the *OsCRTISO* gene of *phs3-1*, *phs3-2*/*NG0489* and *phs3-3* mutants.Expression of the *OsCRTISO* gene in wild type and mutants.The positions of the mutation in the *β-OsLCY* gene of *phs4-1* and *phs4-2* mutants.Expression of the *β-OsLCY* gene in the wild type and mutant. Arrowheads indicate the insertion sites of a rice retrotransposon, *Tos17*. Boxes and lines indicate exons and introns, respectively.

Because only mutants specifically blocked in the carotenoid biosynthetic pathways can manifest as a combination of vivipary with albino phenotype (Wurtzel *et al.*, 2001), we presumed that these mutants may carry lesions in key genes for biosynthesis of carotenoids or ABA. So the predicted amino acid sequences of ABA biosynthesis genes from Arabidopsis were used as probes to screen *in silico* all available rice databases. In these four regions where mutations *phs1* through *phs4* mapped, four candidate genes appearing to be orthologs of Arabidopsis genes were found in the carotenoid biosynthesis pathway (Table 1), including a *PDS*-like gene (*phs1*), a *ZDS*-like gene (*phs2-1* and *phs2-2*), a carotenoid isomerase-like gene (*phs3-1*) and a *β-LCY*-like gene (*phs4-1* and *phs4-2*). To examine whether these candidate genes carried mutations in the respective mutants, we analyzed the corresponding candidate genes by polymerase chain reaction (PCR) and sequencing; the results revealed that a *Tos17* was inserted in intron 4 of the putative *OsPDS* in *phs1* (Figure 2a). As expected, no *OsPDS* expression was detected by reverse transcription (RT)-PCR analysis in *phs1* (Figure 2b). The *phs2-1* carried a *Tos17* insertion in exon 3 of *OsZDS*, whereas *phs2-2* had a 1.7-kb deletion started from intron 3 spanned to exon 7 (Figure 2c). No *OsZDS* expression was detectable in either mutant allele (Figure 2d).

In *phs3-1*, a G-to-C transition was identified at position 1995 (the putative translation start codon is referred to as +1) of the *OsCRTISO* gene, which is predicted to be the donor site of intron 6 (Figure 2e). This mutation presumably causes the utilization of a novel splicing donor-like site (GT) 24 bp upstream from the mutated site. Consistent with this speculation, RT-PCR analysis revealed the transcript in *phs3-1* was slightly shorter than that in the wild type (Figure 2f), which was confirmed by direct DNA sequencing. In addition, another two *phs3* alleles were collected by the *zebra* phenotype. Sequence analysis revealed that mutations of various types were identified in the candidate gene *CRTISO*. A *Tos17* retrotransposon inserted in exon 7 of *phs3-2* (NG0489 from the Rice Tos17 Insertion Mutant database http://tos.nias.affrc.go.jp/~miyao/pub/tos17/index.html.en) completely disrupts the function of *CRTISO* (Figure 2f). *phs3-3* has a 30-bp deletion in exon 6. The deletions in *phs3-1* and *phs3-3* do not cause a frame-shift in the open reading frame (ORF).

The rice *β-OsLCY* gene has no intron, and sequence analysis of *phs4-1* mutant showed a 1-bp deletion at the 1314 bp starting from ATG, and the *phs4-2* allele has a 25-bp deletion in 473–497 bp (the putative translation start codon is referred to as +1). The deletions caused a frame-shift and subsequently created a new stop codon that might result in immature translation (Figure 2g) and disrupt the normal function of the *β-LCY* gene. Expression analysis also showed that the *β-OsLCY* transcript was barely detectable in *phs4* alleles compared with the wild type (Figure 2h).

**Figure 3:**
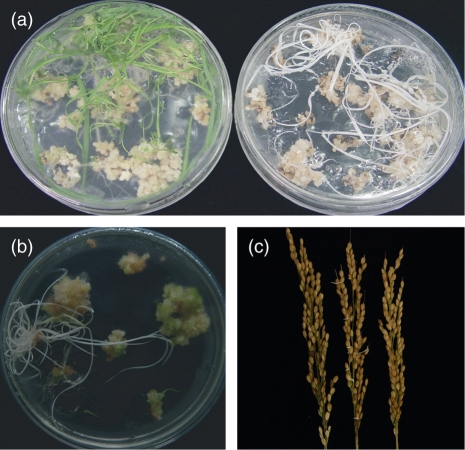
Complementation test.
Plant regeneration from calli derived from embryos of the heterozygous *phs4* or wild-type seeds (left) and the homozygous *phs4* seeds (right).The calli derived from embryos of homozygous *phs4* seeds were used for transformation.The phenotypes of panicles in mature plants of wt (left), heterozygous mutant (middle) and complementation transgenic plant (right).

To confirm the identity of these candidate genes, we also performed genetic complementation experiments. Since homozygous mutant seedlings from three genes (*OsPDS*, *OsZDS*, and *β-OsLCY*) are non-viable the homozygous seeds were screened out simply from the heterozygous lines at an early stage of callus induction using mature embryos. The embryos of the homozygous mutant initiated an albino bud after 2 weeks’ culture on Murashige and Skoog medium (Murashige and Skoog, 1962) with addition of 2 mg l^−1^ 2,4-dichlorophenoxyacetic acid (2,4-d) in the light, and their calli derived from homozygous seeds regenerated the albino shoot during later regeneration (Figure 3a). Genomic DNA fragments covering the respective 5′-upstream regions, entire genes and 3′-downstream regions of respective candidate genes were introduced into corresponding mutants by *Agrobacterium*-mediated transformation. In all cases, the mutant phenotypes were rescued when genomic fragments containing the candidate genes were introduced (Figure 3b,c, and Table S3). In contrast, the transgenic plants containing the empty vectors failed to rescue the *phs* mutants (data not shown).

### Carotenoid profiles of phs mutants

**Figure 4:**
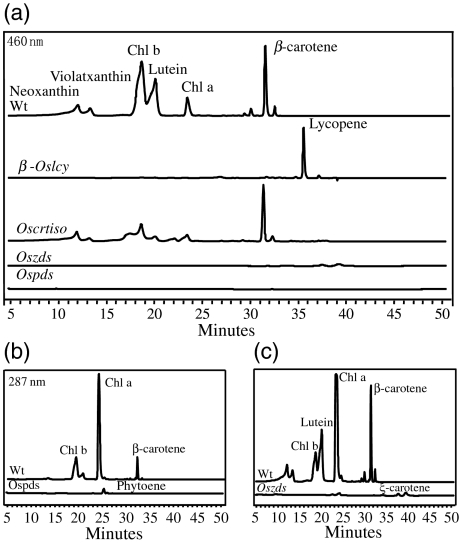
HPLC chromatograms of the wild type and homozygous *phs* mutants grown in light.
HPLC chromatograms recorded at 460 nm (most carotenoids).HPLC chromatograms recorded at 287 nm (phytoene).Chromatograms recorded at 430 nm (ξ-carotene). Chl a, chlorophyll *a*; Chl b, chlorophyll *b*.

To analyze carotenoid metabolism in the *phs* mutants, high-performance liquid chromatograph (HPLC) analysis was performed on the extracts prepared from light-grown wild-type and mutant seedlings. As shown in Figure 4a, carotenoids were almost undetectable in *phs1* (*Ospds* mutants) and *phs2-1* or *phs2-2* (*Oszds* mutants) seedlings at 460 nm. However, lycopene accumulated in *phs4-1* or *phs4-2* (*β-Oslcy* mutants). Carotenoids, especially lutein, were dramatically reduced in light-grown *phs3-1* (*Oscrtiso* mutants) and an all-*trans* lycopene precursor, prolycopene, was accumulated in dark-grown *phs3-1* seedlings (data not shown). The *Ospds* mutant contained a peak at 287 nm with a retention time of 25.54 min, which was absent in the wild type (Figure 4b). The derived spectrum of the peak showed characteristics of phytoene with three main peaks (data not shown). In *phs2* seedlings only small peaks of ξ-carotene and its isomers were detected at 430 nm, which were absent in the wild type (Figure 4c).

These results were consistent with the mutated genes in the biosynthetic pathway of carotenoids, and carotenoid biosynthesis is indeed impaired in these mutants. The albino phenotypes of the *phs* mutants (*Ospds*, *Oszds*, and *β-Oslcy*) were due to chlorophyll and carotenoid deficiency. The pink embryo in *phs4* suggested lycopene accumulation, and the phenotype of *phs3* (*Oscrtiso* mutant) seemed to result from photobleaching.

### Cellular characters of the phs mutants

**Figure 5:**
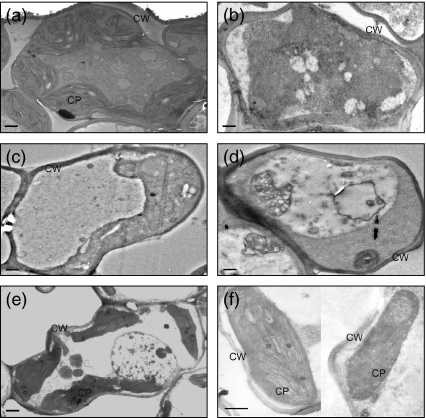
Mesophyll cells and chloroplasts from the *phs* mutants and wild type. Wild type (a), *Ospds* (b), *Oszds* (c), *β-Oslcy* (d), *Oscrtiso* (e); the chloroplast from wild type (left) and *Oscrtiso* mutant (right; f). CP, chloroplast; CW, cell wall; Thy, thylakoid. Scale bar = 1 μm.

Carotenoids are essential components of photosynthetic systems, which occur in thylakoid membranes in chloroplasts in association with chlorophyll–protein complexes (Green and Durnford, 1996; Moise *et al.*, 2005). To assess the effect of the *PHS* mutations on chloroplast development, the leaves of 2-week-old seedlings of all *phs* mutants mentioned above were examined by transmission electron microscopy. In wild types, chloroplasts were well-developed and organized in the mesophyll cells (Figure 5a), in contrast, no plastid- or chloroplast-like structures were found in the mesophyll cells of albino mutants (*phs1*, *phs2*, and *phs4*; Figure 5b–d). In the mesophyll cells of *Oscrtiso* mutants, irregularly shaped and abnormally developed chloroplasts were observed, with reduced and irregularly organized thylakoid membranes (Figure 5e,f).

### OsLCY-RNAi plants and phs mutants suffered from photo-oxidative damages

**Figure 6:**
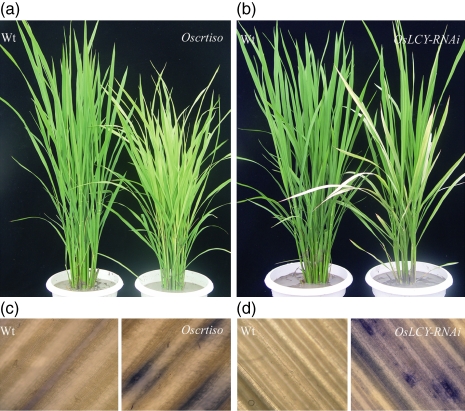
Photobleaching phenotype (a, b) and the detection of ROS (c, d) of *Oscrtiso* and β-*LCY*-RNAi plants and the corresponding wild types.

Due to the photoprotective function of carotenoids, plants deprived of these pigments suffered from photobleaching damage, especially under high light conditions (Niyogi, 1999; Sagar *et al.*, 1988). Since the homozygous mutant seedlings from three genes (*OsPDS*, *OsZDS*, and *β-OsLCY*) are nonviable, we generated transgenic rice plants harboring the *β-OsLCY*- RNAi construct. It was demonstrated that *β-OsLCY-* RNAi plants and *Oscrtiso* mutants showed a distinctly photobleached phenotype on leaves (Figure 6a,b), which was in accordance with the accumulation of ROS as revealed by nitro blue tetrazolium (NBT) staining (Figure 6c,d). Furthermore, compared with that in the wild type, higher activities of ROS-scavenging enzymes were found in both β-*OsLCY*-RNAi plant and *phs3-1* mutant (Figure S3), suggesting that these two plants were under oxidative stress.

**Figure 7:**
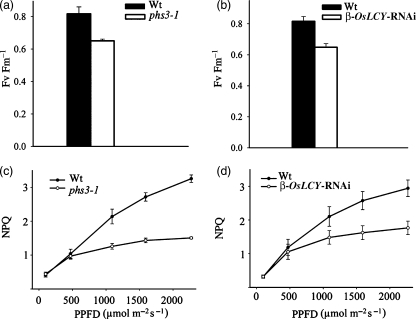
The maximal efficiency of PS II photochemistry (*F*_v_/*F*_m_; a, b) and non-photochemical quenching of chlorophyll fluorescence (NPQ; c, d) of *phs3-1* mutant and β-*OsLCY*-RNAi plants. Standard deviations were obtained from six measurements.

The maximal efficiency of PS II photochemistry (*F*_v_/*F*_m_) is often considered to be an indicator of PS II function (Demmig-Adams and Adams, 1992; Krause and Weis, 1991). Therefore, we further investigated PS II photochemistry in *phs3-1*/*Oscrtiso* mutants and β-*OsLCY*-RNAi plants, as well as the wild type. The *F*_v_/*F*_m_ ratios in wild-type plants are above 0.8. However, *phs3-1*/*Oscrtiso* mutant and β-*OsLCY*-RNAi plants show a decrease in the *F*_v_/*F*_m_ ratio, which is about 0.65 (Figure 7a,b).

The *phs3-1*/*Oscrtiso* mutants and β-*OsLCY*-RNAi plants also show different responses of NPQ to increasing photosynthetic photon flux density (PPFD). When PPFD was <500 μmol m^−2^ sec^−1^, the *phs3-1*/*Oscrtiso* mutants and β-*OsLCY*-RNAi plants showed almost the same value of NPQ in comparison with the wild type. With further increase in PPFD, NPQ increased more rapidly in wild-type plants than in the *Oscrtiso*/*phs3-1* mutants and β-*OsLCY*-RNAi plants, leading to much higher values of NPQ than for the latter two plants (Figure 7c,d).

**Figure 8:**
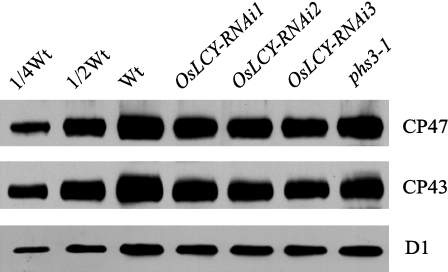
Western analysis of the PS II core proteins CP47, CP43 and D1.

To get further evidence for the photo-oxidative damage occurring in PS II, we examined the levels of essential PS II core proteins CP43, CP47 and D1 (Green and Durnford, 1996), which are the targets of ROS (Niyogi, 1999), in the leaves from the *phs3-1* mutant and β-*OsLCY*-RNAi plants, respectively. Western analysis showed that the level of these proteins in the *phs3-1* mutant and β-*OsLCY*-RNAi plants decreased to 64–77% of that in the wild type, indicating the occurrence of photo-oxidative damage in PS II core proteins (Figure 8).

### ABA and GA synthesis are altered in phs mutants

**Figure 9:**
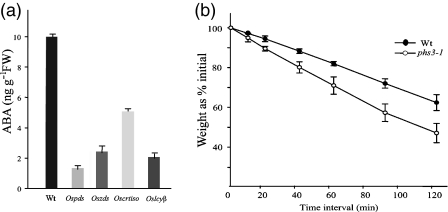
ABA content and water loss assay of *phs* mutants and wild types. (a) ABA content in wild-type and mutant seedlings. (b) Water loss assays for the leaves of the wild type and *Oscrtiso* mutant (T09/*phs3-1*) were performed within 120 min. Standard deviations were obtained from five measurements.

To investigate whether the reduced level of carotenoids in *phs* mutants causes a decreased level of ABA we measured the ABA content in the respective *phs* mutants by immunoassay. As shown in Figure 9a, the amount of ABA was reduced in all four mutants, but much more significantly in *Ospds*, *Oszds*, and *Oslcy* mutants compared with that in the wild type. Since ABA is also directly involved in the regulation of stomatal aperture, we examined the water loss characteristics of *phs3-1/Oscrtiso* plants. Wild-type and mutant plants were grown in the soil under a normal irrigation regime. The water loss analysis was carried out at the tillering stage, and the results revealed a water loss of about 53% of fresh weight within 120 min in *phs* mutants, whereas wild-type leaves showed only a 38% loss, indicating that the rate of water loss in *phs* mutants was faster than in wild-type plants (Figure 9b).

To test whether *phs* mutant can sense or respond to ABA, we cultured seeds [30 days after pollination (DAP)] from *phs3-1* mutant and the wild type in water with or without ABA (of different concentrations) treatment (Figure S4). In all treatments, the *phs3-1* mutant and the wild type grew with similar kinetics, indicating that the *phs3-1* mutant has a normal response to ABA.

**Figure 10:**
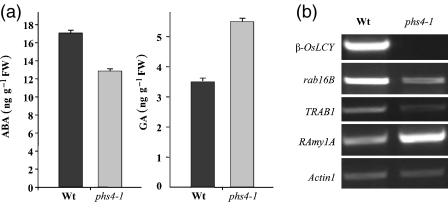
Hormone contents and expression of hormone-regulated genes. (a) ABA and GA content in wild-type and *β-Oslcy* seeds at 30 days after pollination (DAP). (b) Expression of *rab16B* (LEA protein gene), *TRAB1* (a bZIP factor gene) and *RAmy1A* (α-amylase gene) genes in wild-type and *β-Oslcy* seeds at 30 DAP.

Abscisic acid and another important phytohormone, GA, which can directly antagonize ABA signaling during induction of dormancy, are two important regulators of seed dormancy. Therefore, we measured the ABA and GA content in homozygous *phs4-1*/*β-Oslcy* seeds 30 DAP (*phs4* homozygous seeds were easily discernible by their pink embryo). As expected, the ABA level in *phs4-1*/*β-Oslcy* was lower than that in the wild type; however, GA in *phs4-1*/*β-Oslcy* is higher than that in the wild type (Figure 10a). We further examined the expression of two ABA-regulated genes, *rab16B* and *TRAB1*, as well as a GA-induced gene, the α-amylase gene *RAmy1A* (AK073487), which were involved in embryo development and seed maturation (Hobo *et al.*, 1999; Mitsui *et al.*, 1996; O'Neill *et al.*, 1990; Ono *et al.*, 1996). The results demonstrated that the transcripts of *rab16B* and *TRAB1* were significantly reduced in *phs4-1*/*β-Oslcy* seeds while the transcript of *RAmy1A* was increased (Figure 10b), suggesting that the ABA/GA ratio in *phs4-1*/*β-Oslcy* seeds is probably a switch for PHS.

## Discussion

### Analysis of phs mutants in rice

Under normal circumstances, germination in the ear of developing cereal grain is prevented by physiological mechanisms that include dormancy. Only under exceptional circumstances (e.g. rain late in grain maturation) may PHS occur. Since dormancy is a complex trait which is controlled by a large number of genes, not only with strong intergenic interactions but also strong interactions between genes and the environment. Thus, QTL analysis has often been the tool of choice for the dissection of dormancy and germination, and a large number of the QTLs controlling seed dormancy and germination have been identified in Arabidopsis, rice, wheat, and barley (Clerkx *et al.*, 2004; Li *et al.*, 2004; Prada *et al.*, 2004). Comparative genomics approaches were also used to identify candidate gene(s) controlling dormancy and PHS based on the availability of the rice genome sequence, a large number of barley and wheat ESTs, and also the high co-linearity among rice, wheat, and barley (Li *et al.*, 2004; Wilkinson *et al.*, 2002).

Since PHS is a complex trait controlled by both environmental and genetic factors, we carried out intensive screening of the rice mutant population in south China, where long spells of rainy weather often occur in early summer and autumn and successfully isolated 27 rice *phs* mutants in the paddy fields.

In this study, we focused on *phs* mutants which belong to category I; using both map-based cloning and comparative genetics approaches, we have identified that the mutated genes in category I mutants encode major enzymes (*OsPDS*, *OsZDS*, *OsCRTISO*, and *β-OsLCY*) in the carotenoid biosynthesis pathway. The candidate genes were all confirmed via functional complementation. Further work with these rice *phs* mutants will provide novel insights into the role of these *PHS* genes in dormancy and germination.

### Carotenoid metabolism, plastid development and photo-oxidation in phs mutants

In plant chloroplasts, carotenoids serve important roles in the photosynthetic apparatus as structural components and photosynthetic and photoprotective pigments (Green and Durnford, 1996; Niyogi, 1999; Niyogi *et al.*, 2001). Just as expected, according to the enzymatic steps blocked, *phs1* and *phs2* accumulated phytoene and ξ-carotene in the light-grown seedlings, respectively. The results are in accordance with maize *vp5* and *vp9* mutants with mutations in *PDS* and *ZDS* (Hable *et al.*, 1998; Matthews *et al.*, 2003). In case of *phs4*, the mutation in lycopene β-cyclase leads to high lycopene accumulation in both seedlings and embryos from homozygous *phs4* seeds. The accumulation of lycopene may be explained by the presumption that lycopene ε-cyclase and lycopene β-cyclase constitute an enzyme complex in the thylakoid membranes of plant chloroplasts; in the absence of β-cyclase, the complex is destabilized, resulting in the loss of both ε- and β-cyclase activity and so lycopene accumulates (Cunningham and Gantt, 1998). The results are consistent with 2-(4-chlorophenylthio)-triethylamine hydrochloride (CPTA)-treated barley seedlings (CPTA is a specific inhibitor of the cyclization of lycopene, especially lycopene β-cyclase; La Rocca *et al.*, 2007). In addition, *PSY* mRNA is virtually absent in rice endosperm, which does not provide the substrate for these downstream enzymes and is consequently unable to form downstream products (Schaub *et al.*, 2005). Therefore, the *phs4* mutant accumulates lycopene (showing a pink color) in the embryo but not in the endosperm.

Unlike *phs1*, *phs2*, and *phs4* mutants, *phs3* mutant show a non-lethal and ‘variegated’ phenotype. The mutation in the *PHS3* gene results in a dramatic reduction of lutein in light-grown leaves and accumulation of prolycopene in etiolated seedlings. This result was in agreement with other *crtiso* mutants from tomato and Arabidopsis (Isaacson *et al.*, 2002; Park *et al.*, 2002). Interestingly, the CRTISO activity could partially be substituted by light, i.e. photoisomerization, which may account for the survival of the *phs3* mutant (Isaacson *et al.*, 2002; Park *et al.*, 2002).

Since carotenoids are located in the photosynthetic membrane in the form of chlorophyll–carotenoid–protein complexes and some carotenogenic enzymes are membrane-associated (Cunningham and Gantt, 1998; Green and Durnford, 1996), changes in carotenoid composition or the carotenogenic enzyme itself always lead to abnormal plastid development (Park *et al.*, 2002; Welsch *et al.*, 2000). It has been demonstrated that the location of PSY protein influenced the formation of PLBs (Welsch *et al.*, 2000), CRTISO is required in PS II assembly in cyanobacteria, and loss of CRTISO activity results in the absence of PLB in the *ccr2* Arabidopsis mutant (Masamoto *et al.*, 2004; Park *et al.*, 2002). All the *phs* mutants (*phs1* through *phs4*) showed dramatic changes in carotenoid composition and had abnormal chloroplasts or even none at all, suggesting the importance of these enzymes for plastid development.

Carotenoids could also act as antioxidants to quench excessive free radicals and ROS generated from photo-oxidation (Hirayama *et al.*, 1994; Niyogi, 1999; Niyogi *et al.*, 1998; Woodall *et al.*, 1997). The increasing activities of ROS-scavenging enzymes in *β-OsLCY* RNAi plants and *Oscrtiso* mutants suggested that these two plants suffered from oxidative damage. Non-photochemical quenching of chlorophyll fluorescence is one of the major indicators which are closely associated with the onset of harmless dissipation of the excess energy present in the pigment of light-harvesting complexes as heat (Gilmore, 1997; Niyogi, 1999). The significant decrease of NPQ in *β-OsLCY* RNAi plants and *Oscrtiso* mutants indicated that excessive absorbed light could not be efficiently dissipated as heat and the excessive energy might result in ROS generation. Nitro blue tetrazolium staining further confirmed that considerable superoxide accumulated in both *β-OsLCY* RNAi plants and *Oscrtiso* mutants. The value of *F*_v_/*F*_m_ is normally in the range of 0.8–0.85 in healthy leaves independent of plant species, and a lower value indicated that a proportion of PS II reaction centers were damaged (Demmig-Adams and Adams, 1992). The decreased *F*_v_/*F*_m_ indicated that photoinhibition occurred in the *β-OsLCY* RNAi plants and *Oscrtiso* mutants under normal conditions. Moreover, the decrease of PS II core proteins such as CP43, CP47, and D1 in *β-OsLCY* RNAi plants and *Oscrtiso* mutants may provide further evidence for the occurrence of photo-oxidative damage in PS II. Taken together, these results further confirm that the investigated *PHS* genes are involved in not only synthesis of the carotenoid precursors of ABA, but also chloroplast development and photoprotection. However, in the dark or in weak light the mechanism by which carotenoid deficiency influences plastid development needs to be further elucidated.

### ABA and pre-harvest sprouting in phs mutants

Seed dormancy and germination are regulated by a wide range of plant hormones, including ABA, ethylene, GA, and brassinosteroids, of which ABA is the primary mediator of seed dormancy (Koornneef *et al.*, 2002). Abscisic acid-deficient or ABA-insensitive Arabidopsis mutants show reduced seed maturation and dormancy (Finch-Savage and Leubner-Metzger, 2006; Koornneef *et al.*, 2002; Leon-Kloosterziel *et al.*, 1996b). Unlike in Arabidopsis, in cereal plants such as maize embryos from ABA-deficient mutants germinate precociously on the ear. However, the absolute level of ABA in the seeds does not always perfectly correlate with the dormancy and germination events, suggesting that other modulating factors are also involved (White *et al.*, 2000). It has been presumed that dormancy released by after-ripening and stratification caused a switch to ABA catabolism resulting in a decrease in ABA content in the embryo and a corresponding increase in inactive ABA metabolites (Gubler *et al.*, 2005).

The amount of GA also plays an important role in controlling seed dormancy and germination. Previous studies revealed that paclobutrazol treatment of the ear suppresses vivipary in ABA-deficient seeds (White and Rivin, 2000). Here, we have demonstrated that the impaired biosynthesis of the carotenoid led to significant reduction of ABA content in the *phs* mutants. However, these mutants have a normal response to ABA. Interestingly, the amount of GA in *phs4-1* seeds was significantly increased; the increased GA might result from a reduced flux of GGPP to carotenogenesis since GGPP is the common precursor for both GA and carotenoid biosynthesis (Rodriguez-Concepcion *et al.*, 2001). It has been demonstrated that the level of GA in *PSY*-overexpressing plants was reduced due to increasing utilization of GGPP for carotenoid biosynthesis, leading to a dwarf phenotype (Fray *et al.*, 1995). In addition, the ratio of ABA/GA is distinctly reduced in *phs4-1* seeds, and the genes involved in seed development were differentially expressed in the seeds of the wild type and mutants. These results suggested that the ABA/GA ratio might play an important role in controlling PHS. However, the contribution of carotenoid synthesis to the regulation of balance between ABA and GA is still elusive.

It was found that some common factors regulated ABA and GA biosynthesis during seed development in Arabidopsis in an opposite manner (Gazzarrini *et al.*, 2004). The viviparous mutants of rice are ideal for elucidating the complex mechanism of germination and dormancy, and a detailed comparison on differential expression patterns of genes between different kinds of *phs* mutants and wild type by microarray or other technologies will finally help us to identify the major factors controlling PHS and gain more insight into the physiological functions of carotenoids and the molecular mechanism of PHS.

## Experimental procedures

### Plant materials and growth conditions

Approximately 160 000 rice T-DNA/Tos17 mutant lines (Nipponbare background) were sown in the field in Hangzhou, Zhejiang Province in 2003. The panicles of mature plants were observed in late September to detect the germinated seeds. In 2004, the screened *phs* mutants (T_2_) were sown in Beijing for further screening. Segregation ratios of viviparous and non-viviparous plants were investigated in the T_2_ plants. The other two *phs3* alleles were obtained as follows: *phs3-2* was obtained from TOS17 retrotransposon insertion lines (http://tos.nias.affrc.go.jp/~miyao/pub/tos17/phenotype/17-NG.html, line number NG 0489) and *phs3-3* was derived from a spontaneous mutation.

### Map-based cloning of PHS genes

To map the mutated genes, linkage analyses were performed using an F_2_ population derived from the cross between mutants (Nipponbare, *Japonica*) and TN1 or Minghui 63 (*Indica*) varieties. The simple-sequence repeat (SSR) and sequence-tagged-site (STS) markers used to analyze the polymorphisms between Nipponbare and TN1 (or Minghui 63) were obtained from the DNA bank at the National Institute of Agrobiologica Sciences (http://www.nias.affrc.go.jp).

### Sequence analysis

The protein sequences of ABA biosynthetic enzymes from Arabidopsis (http://www.arabidopsis.org/) were compared with the rice database of TIGR Rice Databases (http://www.tigr.org), the Rice Genome Research Program (http://rgp.dna.affrc.go.jp/), the Beijing Genomics Institute (http://btn.genomics.org.cn/rice), and the Knowledge-based Oryza Molecular biological Encyclopedia (KOME; http://cdna01.dna.affrc.go.jp/cDNA/) for searching the rice orthologs.

### Construction of complementation and RNAi vectors and plant transformation

A 6.5-kb DNA fragment containing a full-length genomic *β-OsLCY* gene was obtained by digesting the bacterial artificial chromosome (BAC) clone OSJNBb0031B09 with *Pst* I and *Bam* HI; the fragment was inserted into a binary vector pCAMBIA2300. Similarly, the complementation vectors for other *phs* mutants were also constructed, using corresponding BAC clones (Table S3). In addition, empty vectors were transformed into the corresponding *phs* mutant as the respective controls. For *β-OsLCY* RNAi construct, an 856-bp fragment from 630 to 1486 bp of the *β-OsLCY* ORF was inserted as a *Bam* HI/*Sal* I fragment in sense orientation downstream of the potato (*Solanum tuberosum* L.) GA20 oxidase intron into pUC-RNAi (Luo *et al.*, 2006). The same fragment was inserted in antisense orientation into the *Bgl* II/*Xho* I sites of pUC-RNAi already carrying the sense fragment. Subsequently, the fragment comprising sense and antisense fragments of *β-OsLCY* interspersed by potato GA20 oxidase intron was excised from pUC-RNAi using the flanking *Pst* I and inserted into a pXQAct plasmid between rice *actin1* promoter and *Ocs* terminator, yielding the binary construct. The rice transformation was performed as described (Liu *et al.*, 2007).

### Carotenoid analysis

The extraction and analysis of carotenoids was performed as previously described (Fraser *et al.*, 2000). Briefly, a reverse-phase C30, 5 μm column (250 × 4.6 mm) coupled to a 20 × 4.6 mm C30 guard (YMC Inc.; http://www.ymc.co.jp) with a methanol/*tert*-methyl butyl ether-based mobile phase were used with a HPLC 10Avp system (Shimadzu, http://www.shimadzu.com/). Throughout chromatography, the elution was monitored continuously from 200 to 600 nm by an online Shimadzu SPD-10Avp PDA detector. Carotenoids were identified by their characteristic absorption spectra, typical retention time, and comparison with authentic standards.

### Transmission electron microscopic analysis

Rice leaves were cut into 1 mm squares, fixed in 2.5% (v/v) glutaraldehyde in PBS (pH 7.2) for 24 h and post-fixed in 2% OsO_4_ in PBS (pH 7.2) for 2 h. Following ethanol series dehydration, samples were embedded in Epon 812 (Shell Chemicals; http://www.shellchemicals.com) and polymerized for 24 h at 60°C. Ultrathin sections (50–70 nm) were double stained with uranyl acetate and lead citrate and examined with a transmission electron microscope (TEM; FEI Tecnai 20; http://www.fei.com) at 120 kV.

### Histochemical detection of O_2_^−^

Superoxide accumulation in rice leaves was visualized by 0.1% NBT staining as described (Fitzgerald *et al.*, 2004). Rice leaves were vacuum-infiltrated and stained with NBT solution. After staining, the chlorophyll was removed by incubating in 96% (v/v) ethanol overnight.

### Chlorophyll fluorescence measurements

Chlorophyll fluorescence measurements were carried out with attached leaves in the greenhouse using a PAM 2100 portable fluorometer (Walz, http://www.walz.com/) as described (Lu *et al.*, 2001).

### Determination of ABA, GA, and water-loss assay

Determination of ABA and GA with 2-week-old seedlings was performed as previously described (Agrawal *et al.*, 2001; Luo *et al.*, 2006). For the water-loss assay, plants were grown in a paddy field and the detached leaves (300 mg from 10 seedlings) of 1-month-old seedlings were kept on aluminum foil at room temperature (25°C). The weight of leaves was taken at 10-min intervals until 60 min or longer. Each experiment was performed with five replicates.

### Germination and ABA response assay

Seeds (30 days after pollination) from *phs* mutants, the wild type and complementation transgenic plants were sampled for germination ratio and ABA response assay. Seeds were cultured in a growth chamber at 30°C with a 16-h/8-h cycle for 3 days for the germination assay and 11 days for the ABA response assay. Shoots from seeds without ABA treatment were measured regularly, while shoots from seeds treated with different concentrations of ABA were measured at the 11th day after growth.

### Expression analysis

Total RNAs were isolated from various organs of rice as described (Luo *et al.*, 2006). The RT-PCR was performed with DNase-treated total RNAs using the RT-for-PCR Kit (Promega, http://www.promega.com/). The primers used for each gene are listed in Table S1. The products of PCR amplification were separated by electrophoresis on 1.5% (w/v) agarose gels and stained with ethidium bromide.

### Western blot analysis

Thylakoid were extracted from the first leaves of β-*OsLCY*-RNAi plants, *phs3-1* mutant and the wild type when β-*OsLCY*-RNAi plants and *phs3-1* mutant showed a photobleaching phenotype at the tillering stage. Thylakoid membrane preparation and immunodetection of the PS II core proteins CP43, CP47 and D1 were carried out as described (Peng *et al.*, 2006).

### Enzyme activity assay

All enzyme activities were measured from 0.5 g first leaves of β-*OsLCY*-RNAi plants and the *phs3-1* mutant as well as the wild type when β-*OsLCY*-RNAi plants and the *phs3-1* mutant showed a photobleaching phenotype at the tillering stage. The enzyme activities of SOD, CAT, APX, MDAR, DHAR, and GR were measured as described previously (Chen and Gallie, 2004; Gonzalez *et al.*, 1998; Jiang and Zhang, 2002; Knöraer *et al.*, 1996).

## Supplementary Material

The following supplementary material is available for this article online:

Figure S1 Carotenoid and abscisic acid biosynthetic pathway.

Figure S2 The phenotypes of the rice pre-harvest sprouting mutants.

Figure S3 Activities of ROS-scavenging enzymes of leaves from *OsLCY-RNAi* plant, *phs3-1*, and wild type at tillering stage.

Figure S4 Abscisic acid responsiveness test of *phs3-1* mutant.

Figure S5 Map-based cloning of *PHS* genes.

Table S1 The primers used for expression analysis via RT-PCR.

Table S2 Segregation ratios of viviparous and non-viviparous plants in the T2 generation.

Table S3 Constructions for genetic complementation of *phs* mutants and plant transformation events.

This material is available as part of the online article from http://www.blackwell-synergy.com.
