# Supplementary material for: Mutations of genes in synthesis of the carotenoid precursors of ABA lead to pre-harvest sprouting and photo-oxidation in rice
Source: Plant J. 2008 Apr 1;54(2):177–89. doi: 10.1111/j.1365-313X.2008.03411.x (PMC2327239; doi:10.1111/j.1365-313X.2008.03411.x)

Supplementary Figures

Supplementary Figure 1

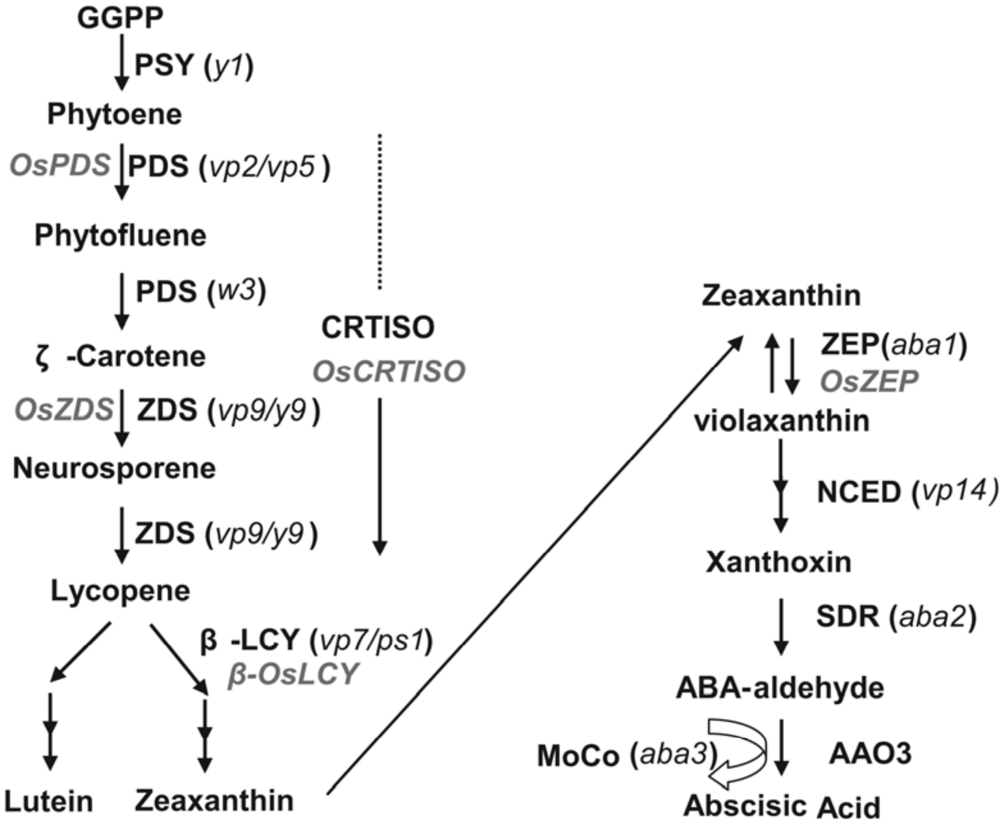

Supplementary Figure 2

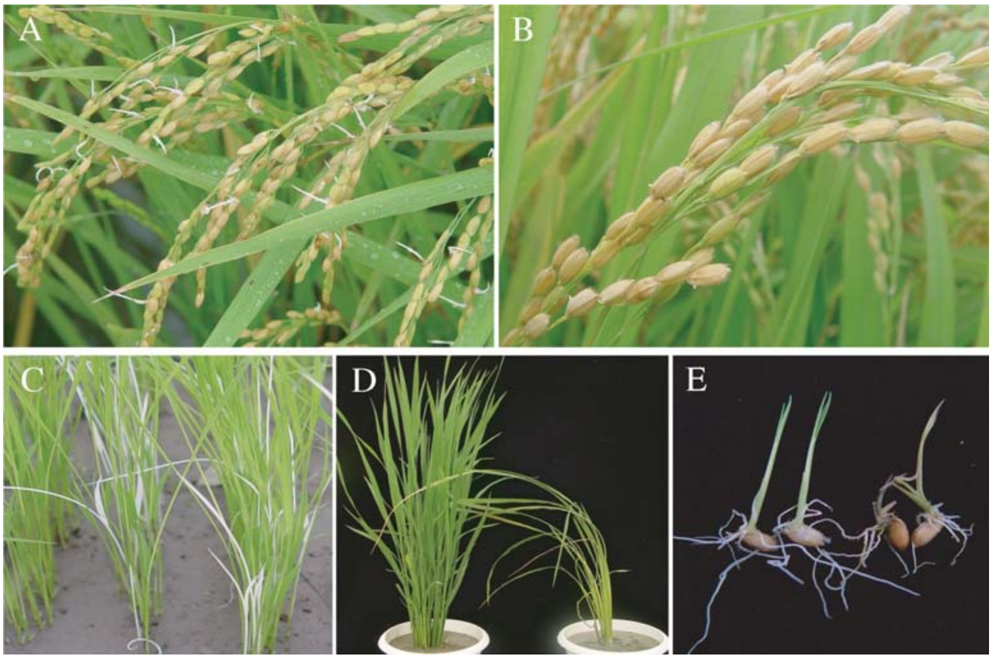

Supplementary Figure 3

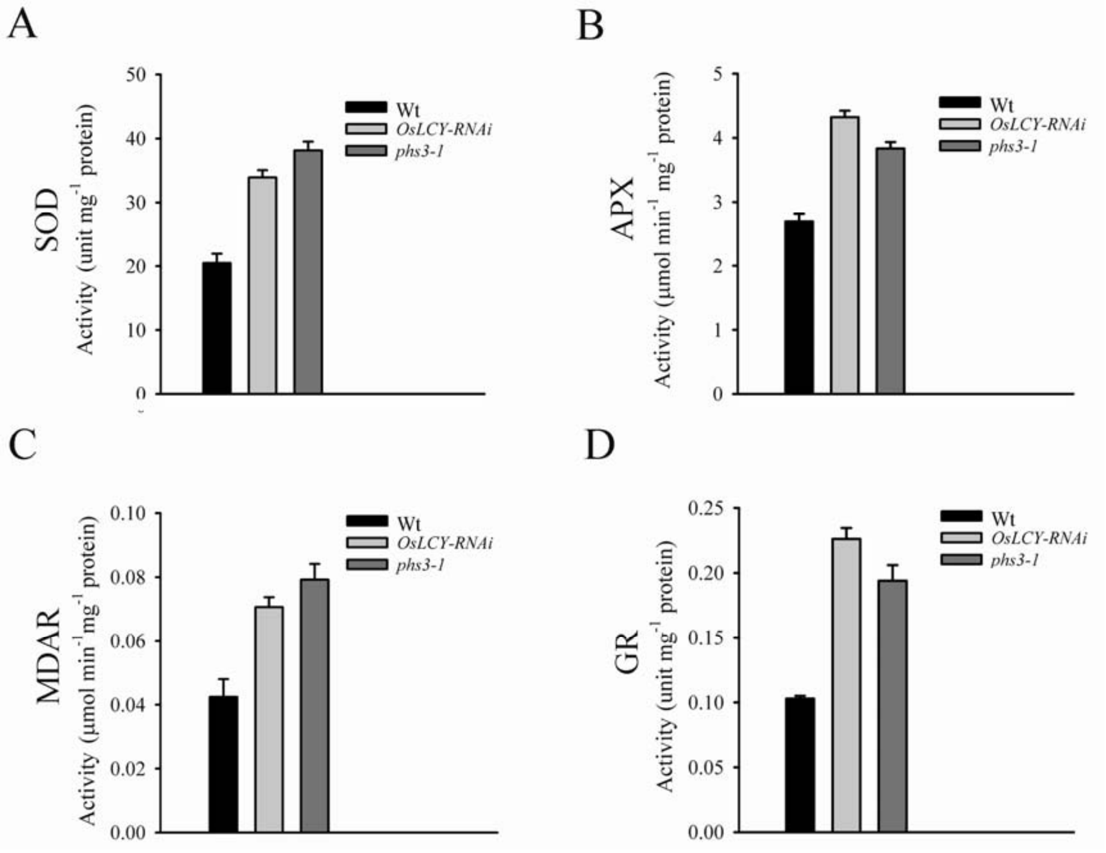

Supplementary Figure 4

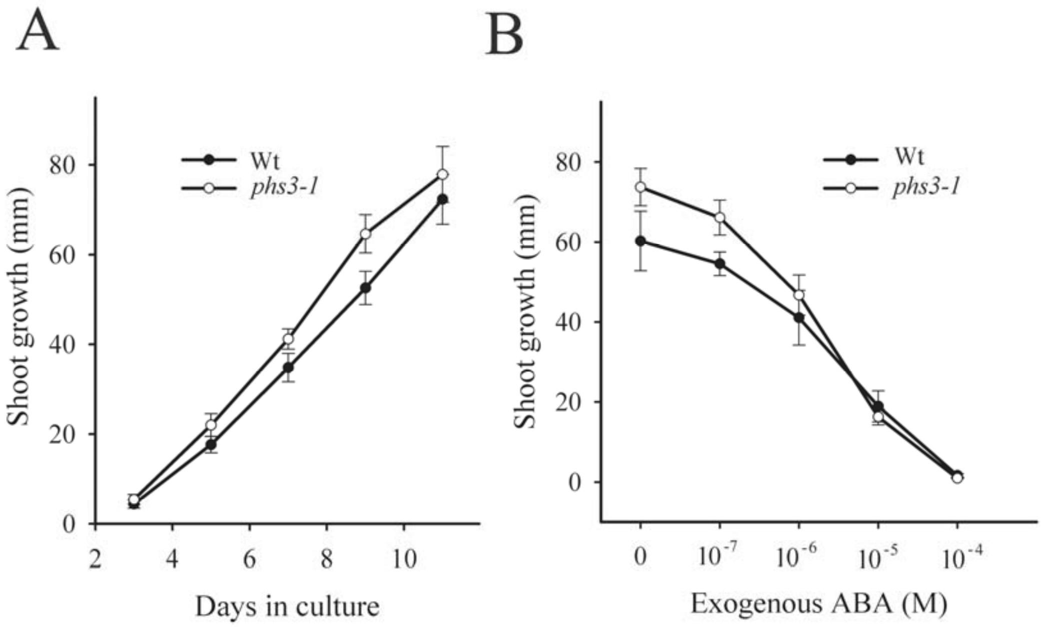

Supplementary Figure 5

A. *phs1*×TN1 (n =280)

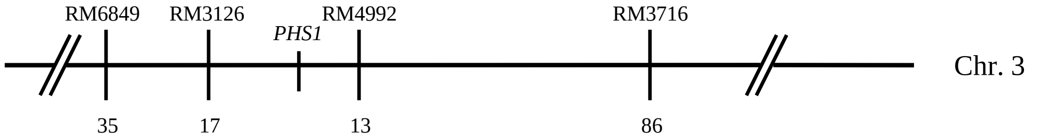

B. *phs2-1*×TN1 (n = 350)

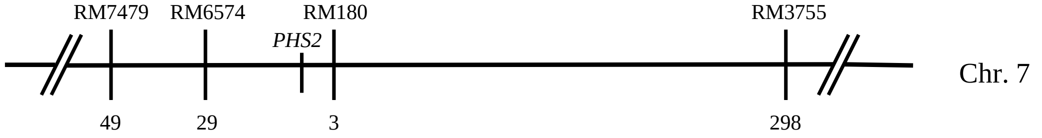

C. *phs3-1*×Minghui 63 (n = 350)

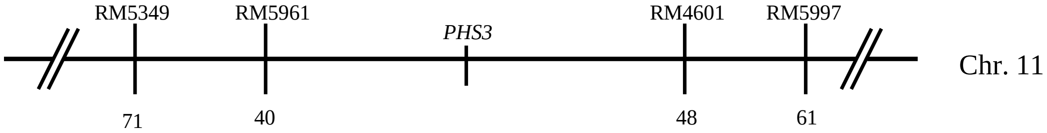

D. *phs4-1*×TN1 (n = 400)

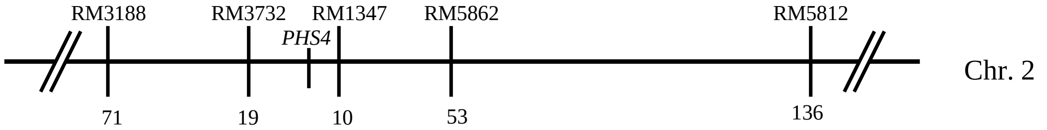

Supplement: Figure S1 — Carotenoid and abscisic acid biosynthetic pathway. [file tpj0054-0177-SD1.pdf]
