# Supplementary material for: Mutations of genes in synthesis of the carotenoid precursors of ABA lead to pre-harvest sprouting and photo-oxidation in rice
Source: Plant J. 2008 Apr 1;54(2):177–89. doi: 10.1111/j.1365-313X.2008.03411.x (PMC2327239; doi:10.1111/j.1365-313X.2008.03411.x)
Supplement: Table S1 — The primers used for expression analysis via RT-PCR. [file tpj0054-0177-SD6.doc]

**Supplementary Tables**

Supplementary Table S1 The primers used for expression analysis via RT-PCR.

| Gene | Forward primer | Reverse primer | PCR product |
| --- | --- | --- | --- |
| *OsPDS* | tcctcttgtttttgcagacg | atttaagggtgcaggcaatg | 717bp |
| *OsZDS* | aatggaggcaatgggataca | ccagcatgtgtcatttgagg | 668bp |
| *β-OsLCY* | cctcgtccagtacgacaagc | atgcagatcctcacctcacc | 856bp |
| *OsCRTISO* | CCGTCATGTTCGGCTTCTC | AAACTGAGGCTTTGACACCC | 815bp |
| *rab16B* | TTGCTTGAGAGGATGGACAA | CCATGAATCCCTTCTTCTCG | 471bp |
| *TRAB1* | TTAGAGGCGGTCAACAATCC | CGTGTCGGCAAACTACAAAA | 386bp |
| *RAmy1A* | AGCTGGTGAACTGGGTGAAC | GCGGTTATCTCCTGCTTCAG | 322bp |
| *Actin1* | AGCAACTGGGATGATATGGA | CAGGGCGATGTAGGAAAGC | 434bp |

Supplementary Table S2 Segregation ratios of viviparous and non-viviparous plants in the T2 plants.

| Original line | progenies of segregated plants^1)^ | | | P |
| --- | --- | --- | --- | --- |
|  | VP | NVP | X^2^(2:1) |  |
| T01 | 120 | 63 | 0.06 | 0.90-0.75 |
| HF807 | 102 | 56 | 0.23 | 0.75-0.5 |
| HG4123 | 82 | 46 | 0.28 | 0.75-0.5 |
| HC2621 | 118 | 61 | 0.02 | 0.95-0.9 |
| HD1449 | 96 | 54 | 0.37 | 0.75-0.5 |
| 1)These plants were derived from the self-pollinated plants that segregated viviparous seeds | | | | |

**Supplementary Table S3 Constructions for genetic complementation of *phs* mutants and plant transformation events**

| Mutants | *phs1* | *phs2-1* | *phs3-1* | *phs4-1* |
| --- | --- | --- | --- | --- |
| Genes | *OsPDS* | *OsZDS* | *OsCRTISO* | *β-OsLCY* |
| BAC/PAC | OSJNBa0032G08 | P0431A02 | OSJNBb0077M24 | OSJNBb0031B09 |
| Restriction site | *Kpn*I and *Bam*HI | *Pst*I | *Kpn*I and *Xba*I | *Pst*I and *Bam*HI |
| Fragment size (kb) | 10.4 | 9.0 | 8.7 | 6.5 |
| Binary vector | pCAMBIA 2300 | | | |
| Number of transformants | 17 | 13 | 25 | 21 |
| Number of complemented lines | 17 | 13 | 23 | 21 |
